# Evolutionary and Developmental Associations of Neural Crest and Placodes in the Vertebrate Head: Insights From Jawless Vertebrates

**DOI:** 10.3389/fphys.2020.00986

**Published:** 2020-08-13

**Authors:** Joshua R. York, Tian Yuan, David W. McCauley

**Affiliations:** ^1^Department of Biology, University of Oklahoma, Norman, OK, United States; ^2^Oklahoma Center for Neuroscience, University of Oklahoma Health Sciences Center, Oklahoma City, OK, United States

**Keywords:** cyclostomes, lamprey, hagfish, CRISPR/Cas9, evo-devo

## Abstract

Neural crest and placodes are key innovations of the vertebrate clade. These cells arise within the dorsal ectoderm of all vertebrate embryos and have the developmental potential to form many of the morphological novelties within the vertebrate head. Each cell population has its own distinct developmental features and generates unique cell types. However, it is essential that neural crest and placodes associate together throughout embryonic development to coordinate the emergence of several features in the head, including almost all of the cranial peripheral sensory nervous system and organs of special sense. Despite the significance of this developmental feat, its evolutionary origins have remained unclear, owing largely to the fact that there has been little comparative (evolutionary) work done on this topic between the jawed vertebrates and cyclostomes—the jawless lampreys and hagfishes. In this review, we briefly summarize the developmental mechanisms and genetics of neural crest and placodes in both jawed and jawless vertebrates. We then discuss recent studies on the role of neural crest and placodes—and their developmental association—in the head of lamprey embryos, and how comparisons with jawed vertebrates can provide insights into the causes and consequences of this event in early vertebrate evolution.

## Introduction

**Figure 1:**
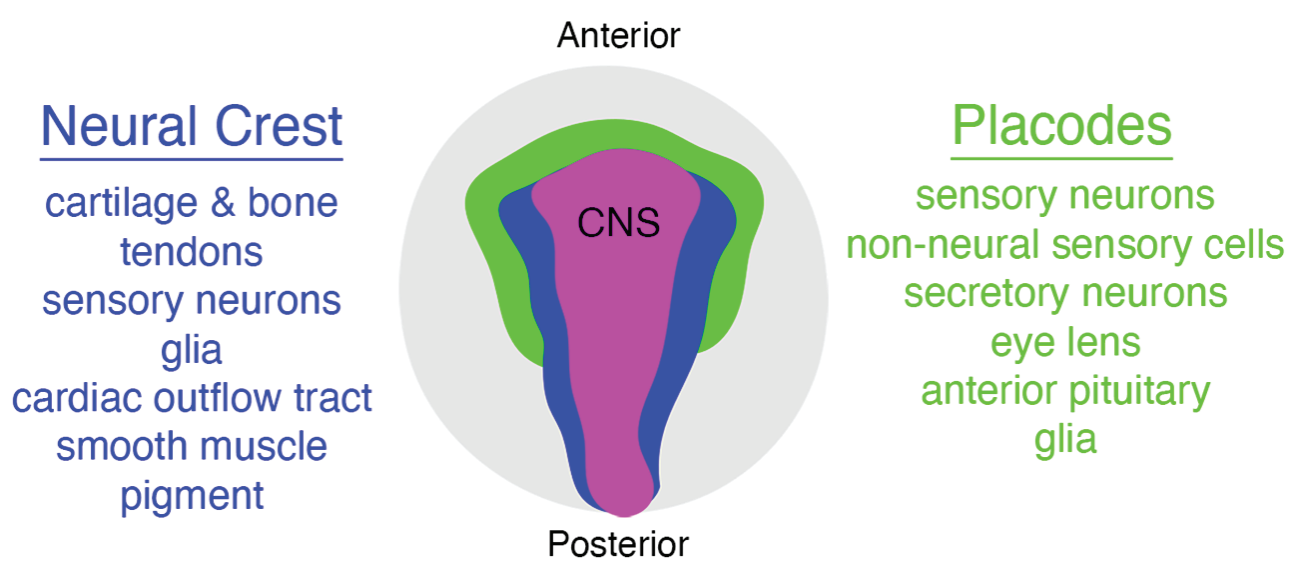
Relationships between neural crest (blue), placode (green), and central nervous system (CNS; pink) populations in vertebrates, with some neural crest and placode derivatives listed on either side. Embryo is viewed from the dorsal aspect.

The vertebrate head is a complex tapestry of morphological features woven together during embryonic development from a varied array of specialized cell types. Although some of the features in the vertebrate head are derived from populations of cells that are evolutionarily ancient, and hence not unique to vertebrates (e.g., mesoderm and endoderm), there are two notable exceptions to this observation—the neural crest and placodes (Gans and Northcutt, 1983; Couly et al., 1993; Santagati and Rijli, 2003; Kuratani, 2008; Square et al., 2016b; Hall, 2018; Kuratani and Ahlberg, 2018; Cheung et al., 2019). Both neural crest cells and placodes are found only in vertebrate animals and they are responsible for constructing many of the traits that uniquely define the vertebrate clade (Figure 1), including the cartilage and bone of the head and jaw skeleton, neurons and glia of the peripheral sensory nervous system, colorful patterns of pigmentation, and much more (Green et al., 2015; Ziermann et al., 2018; Fish, 2019; Martik et al., 2019; Vandamme and Berx, 2019; York and McCauley, 2020b).

Given the developmental and evolutionary significance of neural crest and placodes, it should be no surprise that they have remained some of the most intensively studied and scrutinized populations of cells by vertebrate embryologists since their discovery over 150 years ago (His, 1868; Van Wijhe, 1883; Froriep, 1885; von Kupffer, 1891, 1893; Platt, 1894; Conel, 1942; Damas, 1943; Yntema, 1944). Most contemporary researchers in the fields of neural crest and placode “evo-devo” have directed their efforts and expertise toward the study of either neural crest or placodes in isolation, the inevitable result of specialization that characterizes modern scientific research. But it is important to recognize that the “origin story” of the vertebrates cannot be told from the perspective of either cell population alone. Rather, it was and is the intimate association of both neural crest and placodes in the head of vertebrate embryos that came to distinguish the vertebrates from their invertebrate chordate relatives, a point emphasized by Gans and Northcutt almost four decades ago (Gans and Northcutt, 1983; Northcutt and Gans, 1983). Thus, to understand the origin of the vertebrates is to understand how these cell populations became developmentally and evolutionarily coupled in our earliest vertebrate ancestors.

Here, we review the evolution of the developmental association of neural crest and placodes from the perspective of the jawless (cyclostome or “agnathan”) vertebrate lineage. We describe shared and derived patterns of neural crest and placode development in these animals and compare them to well-studied examples from traditional jawed vertebrate model systems. We then focus on recent work describing the developmental association of neural crest and placodes in the head of jawless vertebrate embryos and how these studies, when placed within a comparative embryology framework, can provide important clues as to how the intimate relationship between these unique cell populations first evolved in early vertebrates.

## Development of Neural Crest and Placodes in Jawed Vertebrates

The jawed, or gnathostome, vertebrates are a monophyletic group that includes representatives of all but two extant lineages of vertebrate animals that diversified from a common ancestor nearly 475 million years ago (Brazeau and Friedman, 2015; Nelson et al., 2016). Jawed vertebrates are comprised of groups such as aquatic “fishes” (quotations denote a paraphyletic assemblage), as well as “amphibians,” “reptiles,” birds, and mammals that share traits including articulated jaws with teeth, paired fins, and paired nasal openings (diplorhiny), among others (Brazeau and Friedman, 2014, 2015). Much of our understanding of the development and evolution of neural crest and placodes has been informed by “traditional” model systems belonging to the jawed vertebrate lineage (e.g., mouse, *Xenopus*, and zebrafish). This is due in part to convenience as many jawed vertebrate models are relatively easy to obtain and rear in standard laboratory conditions, have publicly available and well-annotated genomes and transcriptomes, and are amenable either to the propagation of stable genetic lines and/or modern genome editing, and high-throughput molecular techniques. Below, we describe briefly the developmental mechanisms and genetic control of neural crest and placode development in jawed vertebrates.

### Neural Crest

The neural crest is a migratory, embryonic stem cell population that gives rise to diverse tissues and structures throughout the vertebrate head and trunk, including much of the cartilage and bone of the craniofacial skeleton, melanocytes, many of the sensory neurons and glia of the peripheral nervous system, endocrine cells, as well as tooth and heart primordia (Figure 1; Le Douarin and Kalcheim, 1999; Hall, 2008; Trainor, 2013; York and McCauley, 2020b). Moreover, it has been shown recently that the trunk skeletal tissue in extant cartilaginous fishes such as sharks is also derived from neural crest cells, a feature likely homologous to the body armor of long-extinct fishes such as the “ostracoderms” and “placoderms” (Gillis et al., 2017).

Neural crest cells arise in the neural plate border, a region positioned between the medial neural plate (presumptive central nervous system, CNS) and lateral non-neural ectoderm (comprised of presumptive placodes and epidermis; Le Douarin and Kalcheim, 1999; Hall, 2008; Trainor, 2013). During neurulation, the neural plate borders elevate and fuse at the dorsal midline (i.e., the “crest”) of the neural tube (His, 1868; Le Douarin and Kalcheim, 1999; Hall, 2008; Trainor, 2013). Soon thereafter, these cells delaminate from the neural tube, undergo an epithelial-to-mesenchyme transition (EMT), and then embark on long-distance migrations throughout the head and trunk. In the head, cranial neural crest cells typically migrate in streams of aggregated cells, whereas trunk neural crest cells often migrate as individual cells or small groups of cells (Vega-Lopez et al., 2017; Szabó and Mayor, 2018; Li et al., 2019; Goldberg et al., 2020). After tracking along specific routes throughout the embryo, which are shaped in large part by cell-cell guidance systems, neural crest cells finally reach their destination and differentiate into a specific cell type.

As with any other process in the embryo, neural crest development proceeds by the activities of a gene regulatory network (GRN), a complex and organized set of genetic interactions and intercellular signaling pathways that progressively define the regulatory state of these cells from their earliest stages in the neural plate border to their differentiation into cartilage, bone, neurons, and pigment (Bronner, 2014; Simões-Costa and Bronner, 2015; Williams et al., 2019). The neural crest GRN is a spatial and temporal continuum of gene regulatory interactions from start to finish, and cannot therefore, be broken down into completely separable units for each stage of development. We can, however, recognize and study unique GRN “subcircuits”—a set of common gene regulatory interactions that govern similar mechanisms of neural crest development across highly divergent groups (e.g., mouse and fish; Meulemans and Bronner-Fraser, 2004; Sauka-Spengler et al., 2007; Simões-Costa and Bronner, 2015; Hockman et al., 2019; Parker et al., 2019).

The first of these GRN subcircuits is involved in neural crest induction. This is controlled by intercellular signaling systems that are evolutionarily conserved across metazoans, including members of the *Bmp*, *Wnt*, *Fgf*, and *Delta*-*Notch* families (Milet and Monsoro-Burq, 2012; Simões-Costa and Bronner, 2015). These signaling systems are activated in the neural plate, mesoderm and non-neural ectoderm, and converge on regulatory targets in the neural plate border, such as *Pax3/7*, *Msx1/2*, *Zic1/2*, and *Prdm1* (Milet and Monsoro-Burq, 2012; Pla and Monsoro-Burq, 2018). These so-called neural plate border specifiers in turn regulate expression of neural crest specifiers in the dorsal neural tube (*SoxE*-family, *FoxD3*, *Tfap2a*, *Myc*, *Twist*, *Snai1*/*Snai2*, *Id*, and *EdnrB*), which endow the neural crest with a distinct “molecular anatomy” that enables these cells to detach from the neural tube, undergo EMT and migrate, and generate specific precursors (Martik and Bronner, 2017; Lukoseviciute et al., 2018; Ling and Sauka-Spengler, 2019; Soldatov et al., 2019; Rothstein and Simões-Costa, 2020). Neural crest cell differentiation involves the deployment of gene batteries such as *Sox9*, *Sox5/6*, and *Col2a1* for cartilage, *Sox10*, *Mitf*, and *Tyr* for melanocytes, and *Phox2*, *Ascl*, and *Hand2* for sympathetic neurons (Simões-Costa and Bronner, 2015).

### Placodes

Placodes arise as localized thickenings of ectoderm that in turn give rise to cells that make up many of the sensory components in the vertebrate head, such as cranial ganglia and organs of special sense (Figure 1; Schlosser, 2005, 2006, 2010; Patthey et al., 2014). Although there are slight variations across different jawed vertebrate groups, cranial placodes can be categorized broadly into adenohypophyseal, olfactory, lens, trigeminal (ophthalmic = V1, maxillary = V2, and mandibular = V3 divisions), epibranchial, and lateral line placodes (Schlosser and Northcutt, 2000; Xu et al., 2008; Patthey et al., 2014; Piotrowski and Baker, 2014). All of these, with the exception of the adenohypophyseal and lens placodes, produce various types of sensory and/or secretory cells (Schlosser and Ahrens, 2004; Schlosser, 2006, 2010). In addition to variation in the number and/or types of placodes present in different lineages, some placodes have been lost during evolution, such as that of the lateral line placodes eliminated in amniotes during the water-to-land transition (Schlosser, 2005; Washausen and Knabe, 2018).

Placodes arise in the non-neural ectoderm just lateral to the neural plate border, a region known as the pre-placodal ectoderm (PPE; Saint-Jeannet and Moody, 2014; Moody and LaMantia, 2015). The PPE is shaped like a horse-shoe, which wraps peripherally around the anterior neural plate and neural plate border and subsequently fractures into smaller clusters that represent the progenitors of each placode (Saint-Jeannet and Moody, 2014; Moody and LaMantia, 2015). These progenitors will then undergo invagination and/or delamination before differentiating into various types of sensory cells.

The GRN controlling placode developmental in jawed vertebrates has been less intensively studied than that of neural crest cells, but there are still several important conclusions that can be drawn. Induction of the PPE occurs by some of the same intercellular signaling systems that induce neural crest cells (e.g., *Fgf*, *Bmp*, *Wnt*, and retinoic acid; Baker and Bronner-Fraser, 2001; Lassiter et al., 2014; Singh and Groves, 2016). Placode specification occurs *via Six* and *Eya* (*Six1*, *Six4*, *Eya1*, and *Eya2*) factors, which can be viewed as “master regulators” of placode development, in the sense that they are some of the earliest expressed genes in the PPE, they are often continually expressed throughout development in most placodes, and they are functionally required for placode formation in numerous contexts (Sullivan et al., 2019). For example, in *Xenopus*, several transcriptional regulators of neural crest and placode development expressed in the neural (*Pax3*, *Hairy2b*, and *Zic1*) and non-neural (*Tfap2a*, *Msx1*, *Dlx3*) ectoderm are themselves regulated by *Six1* and *Eya1* (Maharana and Schlosser, 2018). Recent transcriptomic analyses have identified hundreds of putative regulatory targets of *Six1* and *Eya1*, including those involved in production of neural progenitors, such as *Sox2* and *Hes8*, and in sensory cell/neural differentiation *via Ngn1* and *Atoh1* (Riddiford and Schlosser, 2016). Additionally, a handful of transcription factors that are important in development of the ectoderm generally, and neural crest specifically, also have overlapping functions in early placode development (e.g., *Dlx*, *Msx*, *Pax*, *Zic* families, *Tfap2a*, *Gata*, and *Foxi*). Of these, there is evidence that a “*Pax* code” involving *Pax6*, *Pax3/7*, and *Pax2/5/8* may pattern placodes along the anterior-posterior axis (Mansouri et al., 1996; Dahl et al., 1997; Baker and Bronner-Fraser, 2000; Modrell et al., 2014). Finally, cell type differentiation of placodes requires the activity of transcription factors known to regulate neural and sensory cell differentiation in deuterostomes and bilaterians, including homologs of *atonal* (*Ath1* or *Math1* in mouse) and *achaete-scute* (*Ash1* or *Mash1* in mouse), as well as *NeuroD*, *Islet1*, *Phox2a*, *Phox2b*, *Brn3a*, and *Brn3c* (Schlosser, 2006).

## Interactions of Neural Crest and Placodes in the Jawed Vertebrate Head

Neural crest and placodes are both vertebrate novelties, but they are also distinct in several ways. Perhaps the most obvious difference is that neural crest cells are capable of generating both ectomesenchyme (e.g., cartilage and bone) and non-ectomesenchyme (e.g., neurons, glia, pigment, and secretory cells), and form throughout the head and trunk. Placodes, by contrast, can only give rise to non-ectomesenchyme and arise exclusively in the head (Baker and Bronner-Fraser, 1997, 2001; Patthey et al., 2014). Another key difference is that whereas EMT and migration are a *sine qua non* of neural crest development, placodes may instead simply invaginate (e.g., lens, adenohypophyseal, and otic placodes) without migrating far from their site of origin (Schlosser, 2002, 2006, 2010). Finally, although they develop as adjacent cell populations in the ectoderm, neural crest and placodes in most jawed vertebrates, with a few notable exceptions, have relatively divergent GRNs that orchestrate their development, even though they likely share a common evolutionary origin (Grocott et al., 2012; Moody and LaMantia, 2015; Riddiford and Schlosser, 2016; Maharana et al., 2017; Martik and Bronner, 2017; Plouhinec et al., 2017; Horie et al., 2018; Maharana and Schlosser, 2018; Streit, 2018).

**Figure 2:**
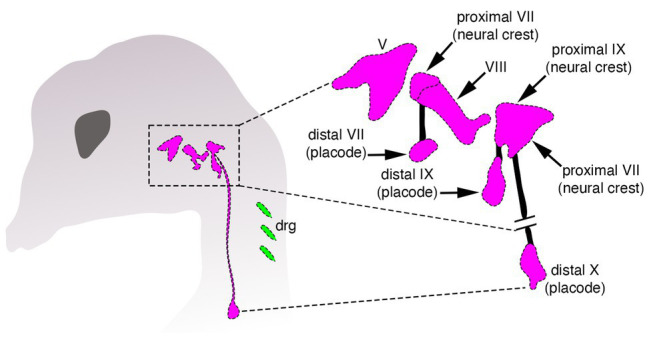
Organization of cranial sensory ganglia in a jawed vertebrate (chicken). Cranial ganglia are pink and dorsal root sensory ganglia (drg) are green. The image on the right shows the distinct proximal and distal ganglion compartments that are formed by neural crest and placodes, respectively, modified from Barlow (2002).

Despite these differences, there is a crucial, but often neglected aspect shared by both neural crest and placodes when discussing the issue of vertebrate origins: these cells work together during development to coordinately generate important structures in the vertebrate head (Grocott et al., 2012; Steventon et al., 2014). The clearest example of this is the creation of the paired sensory ganglia of the cranial peripheral nervous system—structures that are thought to have enabled the transition from passive filter feeding to active predation in early vertebrates (Figure 2; Gans and Northcutt, 1983; Northcutt and Gans, 1983; Northcutt, 2005). Several cranial sensory ganglia are a mosaic of neural crest and placodes, and both cell types must not only be organized together into morphologically and functionally coherent structures, but these structures must in turn form and maintain appropriate connections with the embryonic CNS (Figure 2). For example, placode-derived and neural crest-derived cells in amniotes contribute to distinct proximal and distal components, respectively, of the facial (VII), glossopharyngeal (IX), and vagus (X) nerves (Figure 2; D’Amico-Martel and Noden, 1983; Barlow, 2002; Steventon et al., 2014). In the trigeminal ganglion placodes generate sensory neurons mostly within the distal aspect, whereas neural crest cells produce neurons within the proximal aspect and glia in both aspects (Hamburger, 1961; Lwigale, 2001).

Throughout vertebrate craniofacial development, neural crest and placodes are physically associated, and their mutual interactions coordinate the formation of cranial sensory ganglia (Steventon et al., 2014). During early development, placodes delaminate and migrate slightly earlier than neural crest cells. In cases where both cell populations contribute to ganglia (e.g., epibranchial), earlier-migrating placode cells are usually followed closely behind by cranial neural crest, whereas other placodes, such as the otic, may act as barriers that shape the migratory paths of cranial neural crest originating from the hindbrain *en route* to the pharyngeal arches (Steventon et al., 2014). Although not all cranial neural crest cells contribute to cranial ganglia, there is evidence that they may physically segregate and individuate placode-derived ganglionic clusters during migration, a phenomenon which may be reciprocated by placodes to enable the formation of neural crest streaming in the head (Theveneau et al., 2013; Szabó et al., 2019). These types of intercellular interactions can occur quite early in development, with neural crest and placodes each appearing to be required for the other to undergo migration and morphogenesis of craniofacial structures in a “chase-and-run” model whereby early migrating placodes chemoattract (*via Sdf*) trailing neural crest cells that express the corresponding receptor (*CXCR4*; Theveneau et al., 2010, 2013). Upon physical contact, the neural crest cell then repels the placode cell away. These repeated sets of interactions are thought to bring about the proper migration and shaping of each cell population into ganglia (Steventon et al., 2014).

The close developmental association of neural crest and placodes continues throughout vertebrate craniofacial development, with both modern and classical embryological experiments demonstrating an interdependence of the two populations for proper patterning of the cranial PNS (Steventon et al., 2014). In many of these studies, ablation of the neural crest did not lead to an obvious loss of cranial ganglia *per se*, but rather inappropriate positioning and morphology of the ganglion concomitant with abnormal or absent projections to the CNS (Yntema, 1944; Begbie and Graham, 2001). Additionally, development of distal ganglia can occur in absence of the proximal components (Kuratani et al., 1991). Genetic ablation of the neural crest or perturbation of proper neural crest migration can lead to inappropriate fusions of otherwise physically separated ganglia (Gassmann et al., 1995; Golding et al., 2004; Osborne et al., 2005; Schwarz et al., 2008). Finally, there is evidence that cranial neural crest cells actively form “corridors” that actually guide the migration and orchestrate patterning of sensory neurons derived from placodes (Freter et al., 2013). These results all point to an important role in the interaction of neural crest and placodes to form cranial sensory ganglia in the head of jawed vertebrates.

## Cyclostomes and the Evolutionary Analysis of Neural Crest and Placodes

The accurate reconstruction of ancestral vertebrate conditions, including the developmental association of neural crest and placodes in the vertebrate head, requires the careful choice of study systems within a comparative (evolutionary) framework. Embryological studies of model systems from the jawed vertebrate lineage, no matter how carefully or elegantly done, tell us little about ancestral conditions. To do that requires that we compare developmental mechanisms *between* the two major lineages of vertebrates—the jawed and jawless clades—as well as a suitable outgroup, such as the invertebrate chordates. It is this simple but powerfully informative methodology that allows us to infer how developmental associations between neural crest and placodes evolved in the last common ancestor of vertebrates (Shimeld and Donoghue, 2012; York and McCauley, 2020a).

**Figure 3:**
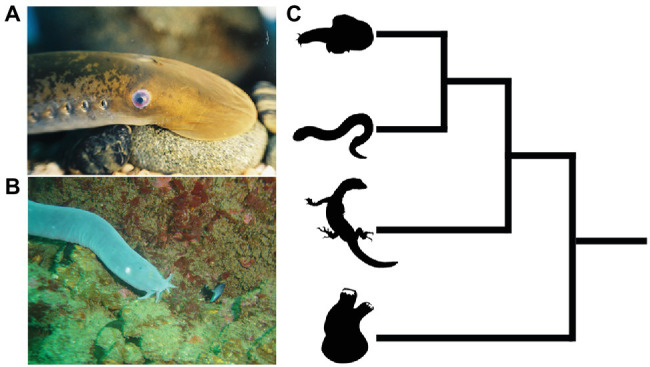
Lampreys **(A)** and hagfish **(B)** are the only extant jawless vertebrates. **(C)** Phylogenetic tree showing relationships among vertebrates and invertebrate chordates. Hagfish and lamprey are on top forming the jawless cyclostome clade, with the jawed vertebrates below. The closest living relatives to the vertebrates are the tunicates, a lineage of invertebrate chordates (bottom). Images from panels **(A)** and **(B)** were used with permission from Wikipedia Commons.

The extant jawless vertebrates, also known as the cyclostomes (Figure 3), are a monophyletic group of animals, and are the sole survivors of a diverse assemblage of jawless fishes that were among the first of their kind to evolve on this planet over 500 million years ago (Hardisty, 1979; Heimberg et al., 2010; Miyashita et al., 2019; York and McCauley, 2020a). They are represented by only two extant groups, the lampreys and hagfishes, which diverged from one another not too long after the cyclostome-gnathostome split. The importance of cyclostomes in understanding vertebrate origins resides in their phylogenetic position as the closest living relatives (i.e., sister group) of the jawed vertebrates (Heimberg et al., 2010). This means that developmental comparisons between jawed and jawless vertebrates allow us, in essence, to work backwards in time and infer how neural crest and placodes became associated in the embryonic head of our vertebrate ancestors, and how subsequent vertebrate lineages elaborated upon these ancestral conditions.

## Development of Lamprey Neural Crest and Placodes

For those interested in comparative vertebrate embryology, lampreys have been the cyclostome study system of choice for quite some time (Green and Bronner, 2014; McCauley et al., 2015; York et al., 2019a). This owes largely to the fact that lamprey adults and their embryos are relatively easy to obtain and rear in simple laboratory settings, at least compared to hagfish (York et al., 2019a; described below). Moreover, accessibility to annotated genomes and transcriptomes, as well as the application of modern molecular genetic techniques such as cell lineage tracing, overexpression of DNAs and RNAs, and knockdown/knockout experiments such as morpholinos and CRISPR/Cas9 genome editing has allowed researchers to address long-standing hypotheses concerning the origin and evolution of vertebrate traits, including neural crest and placodes (McCauley and Bronner-Fraser, 2006; Smith et al., 2013, 2018; Parker et al., 2014; Square et al., 2015; Zu et al., 2016; Hockman et al., 2019; York et al., 2019a; York and McCauley, 2020a).

### Neural Crest

Lampreys, like all other vertebrates, have bona fide neural crest cells. They first arise in the neural plate border and are then brought by neurulation to the dorsal neural tube. From there, they delaminate and undergo an EMT before migrating throughout the head and trunk, where they will eventually reach their target destinations and help generate many morphological features shared with the jawed vertebrates, including a cartilaginous head skeleton, sensory neurons and glia of the peripheral nervous system, and melanocytes (Newth, 1950, 1951, 1956; Nyut, 1955; Langille and Hall, 1988; Horigome et al., 1999; McCauley and Bronner-Fraser, 2003). However, lampreys also lack several of the neural crest-derived structures found in their jawed vertebrate relatives. Included among these are jaws, the myelin sheath surrounding neurons, and sympathetic chain ganglia (Bullock et al., 1984; Shigetani et al., 2002; Häming et al., 2011; Green and Bronner, 2014; Yuan et al., 2018).

The genetic control of neural crest development in lampreys is also very similar to that of jawed vertebrates. The total set of genetic interactions that unfold during neural crest embryogenesis in lampreys, like other vertebrates, is structured into a GRN. This GRN can be broken down into “subcircuits” that direct control of neural crest induction (*Wnt*, *Delta*-*Notch*, and *Bmp* signaling), establishment of the neural plate border (expression of *DlxB*, *Pax3*/*7*, *MsxA*, *ZicA*, and *Prdm1*), as well as specification and migration from the dorsal neural tube (expression of *Snail*, *SoxE1*, *SoxE2*, *Id*, *EdnrB*, *Myc*, *Tfap2a*, *Sip1*/*Zeb2*, *Zeb1*, *type II cadherins*, and many of the neural plate border specifiers; Meulemans and Bronner-Fraser, 2002; Meulemans et al., 2003; Sauka-Spengler et al., 2007; Sauka-Spengler and Bronner-Fraser, 2008; Lakiza et al., 2011; Nikitina et al., 2011; Square et al., 2016a; York et al., 2017). These similarities extend beyond expression patterns. Studies involving gene knockdown/knockout, enhancer analysis, and chromatin profiling have revealed that many of the regulatory interactions at multiple tiers in the neural crest GRN are also shared between lampreys and jawed vertebrates (Sauka-Spengler et al., 2007; Nikitina et al., 2008; Lakiza et al., 2011; York et al., 2017, 2018, 2019b; Hockman et al., 2019; Parker et al., 2019; Yuan et al., 2020). There is also evidence that the production of several neural crest cell types shared between lampreys and jawed vertebrates relies upon a common gene regulatory logic. For example, both groups deploy *Fgf* signaling and *SoxE*-group genes for production of cartilage and melanocytes, and *Phox2* in precursors of neural crest-derived enteric neurons (McCauley and Bronner-Fraser, 2006; Cattell et al., 2011; Lakiza et al., 2011; Jandzik et al., 2014; Green et al., 2017).

### Placodes

Compared to the study of neural crest cells, the body of work on placode development in lampreys has been rather limited. Until recently, almost all of our understanding of placode biology in this group had been limited to a handful of papers describing gene expression patterns, histology, and comparative anatomy. In general, lampreys have homologs of many of the same placodes and placode-derived structures as present in jawed vertebrates, including, olfactory, adenohypophyseal, lens, trigeminal, otic, epibranchial, and lateral line (McCauley and Bronner-Fraser, 2002, 2003; Modrell et al., 2014). There are a few differences between lampreys and jawed vertebrates as well. For example, whereas in jawed vertebrates, the olfactory and adenohypophyseal placodes originate as separate primordia, lampreys have a singular nasohypophyseal placode that forms in the anterior-ventral midline of the head. The fused adenohypophyseal placode in lampreys produces the monorhine state of jawless vertebrates compared to that of diplorhiny in jawed vertebrates, and its separation into separate primordia may have precipitated the evolution of articulated jaws in gnathostomes (Murakami et al., 2001; Oisi et al., 2013b). Another difference, revealed by fate-mapping experiments, was that the separate upper lip and lower lip (velum) innervation patterns by neurons of trigeminal maxillomandibular (mmV) origin in the lamprey mouth may result from these placodes arising as distinct primordia early in development (Modrell et al., 2014).

The developmental mechanisms underlying formation of the PPE in lampreys are almost entirely unknown, with the exception of *DlxB* expression uniquely defining this region, along with overlapping expression of *MsxA* and *Tfap2a*, among others (Sauka-Spengler et al., 2007). It is unknown if the placode specification factors *Six1/2* and *Eya* are expressed in the lamprey PPE, making basic comparisons of early placode development between jawed and jawless vertebrates difficult. Similarly, almost nothing is known regarding the early delamination and migration patterns of placodes in lampreys and how this relates to early neural crest migration.

In terms of functional genetics, knockdown or knockout of early placode specification factors such as *Six*, *Eya*, and *Dlx* have not been performed in lampreys. Again, this leaves considerable uncertainty surrounding the conservation and divergence of gene regulatory interactions orchestrating placode development across vertebrates. Recent work on *Snail* has revealed an early role for this transcription factor during placode development in lamprey. It was found that a single *Snail* ortholog in lamprey was expressed simultaneously in the neural plate border and PPE (York et al., 2019b). There is also evidence that *Snail* is essential for early placode development because CRISPR/Cas9 knockout of *Snail* leads to near-total loss of *DlxB* expression in the pre-placodal domain, with subsequent elimination of placode-derived elements of cranial sensory ganglia that express *Six1/2*, *Pax3/7*, and *Phox2* (York et al., 2019b).

Later during lamprey craniofacial development, the combinatorial expression of several placode markers suggests a high degree of evolutionary conservation across vertebrates. For example, like jawed vertebrates, lamprey placode derivatives express multiple *Pax* genes in the form of a “*Pax* code” along the anterior-posterior axis, with orthologs of *Pax6* expressed in the lens, olfactory, and nasohypophyseal placodes, *Pax3/7* expressed in the ophthalmic division of the trigeminal placode, and *Pax2/5/8* expressed in otic, posterior lateral line, and epibranchial placodes (Murakami et al., 2001; McCauley and Bronner-Fraser, 2002; Modrell et al., 2014). Combinatorial expression of *Dlx* cognates is observed in some placodes as well with *DlxA*, *DlxB*, and *DlxC* in the otic vesicle and *DlxA*, *DlxC*, and *DlxD* in the nasohypophyseal placode (Cerny et al., 2010; Kuraku et al., 2010). Similar to that described in multiple jawed vertebrate model systems, lampreys express orthologs of *Six1/2* in the otic vesicle, posterior lateral line, and epibranchial placodes and *Phox2* in epibranchial ganglia, which are presumably derived in part from placodes (Häming et al., 2011; Green et al., 2017; Hockman et al., 2017; York et al., 2019b).

## Development of Hagfish Neural Crest and Placodes

Compared to lampreys, hagfishes are much more difficult to work with, especially within the context of comparative embryology. Hagfishes live in relatively deep waters and have an obscure reproductive physiology. Moreover, work from the past 100 years has shown that it is no simple matter to culture them in the laboratory (Holland, 2007; Ota and Kuratani, 2008). Consequently, much of our knowledge of hagfish embryology has historically been limited to descriptive embryology. Although advances in laboratory culture methods have enabled a critical re-examination of hagfish development, the unusually slow development of hagfishes has restricted molecular analysis of hagfish embryology to routine gene expression analysis by *in situ* hybridization. Work over the past several decades has revealed that hagfish have neural crest cells and placodes as other vertebrates do and that the developmental mechanisms and regulatory gene expression patterns are reminiscent of what has been described in lampreys and jawed vertebrates (Ota et al., 2007; Kuratani and Ota, 2008).

### Neural Crest

Early investigations into hagfish embryology raised doubts concerning whether or not the development of neural crest cells in these animals was similar to that described in other vertebrates. For example, Conel (1942) suggested that the hagfish neural crest may arise as epithelial pouches that did not delaminate and migrate as mesenchyme, a result which, if confirmed, would suggest a very different route taken by hagfish in the development of this important cell population (Conel, 1942; Ota et al., 2007; Kuratani and Ota, 2008). The matter was settled in 2007 when a report described neural crest development in hagfish as being more or less identical to that described in other vertebrates. Hagfish neural crest cells arise in the dorsal neural tube, express a common suite of neural crest regulatory genes (e.g., *SoxE* and *Pax3/7*), and delaminate and migrate throughout the head and trunk (Ota et al., 2007). Hagfish also share with other vertebrates structures which are presumably derived from neural crest cells, including elements of the cartilaginous head skeleton and cranial sensory and dorsal root ganglia (Wicht and Northcutt, 1995; Braun and Northcutt, 1997; Zhang and Cohn, 2006; Ota et al., 2007; Oisi et al., 2013a). However, despite the overall similarity in neural crest development between hagfish and other vertebrates, there is still much to be learned, an issue that will be difficult to overcome because of the lengthy and complicated development of these animals. Standard techniques in the developmental biologist’s toolkit, including long-term cell lineage tracing and gene knockdown/knockout, are not feasible and this obviously limits the scope of investigation into the developmental genetics of hagfish neural crest and placodes (see below).

### Placodes

Descriptive embryology of the hagfish head has suggested that there are several cranial placode primordia, including, among others, those of epibranchial, otic, trigeminal, lens, and lateral line origin, as well as a singular adenohypophyseal placode as observed in lampreys (Wicht and Northcutt, 1995; Braun and Northcutt, 1997; Wicht and Tusch, 1998; Oisi et al., 2013b). The placode primordia in hagfishes seemingly form as a contiguous, horseshoe-shaped PPE that encompasses the domains from which peripheral cranial nerves will emerge, although it is difficult to say if individual placodes can be observed in isolation early in development as in gnathostomes (Schlosser, 2005, 2017; Ota and Kuratani, 2007; Oisi et al., 2013b). Sensory innervation by some placodes has been described for the trigeminal, facial, glossopharyngeal, and vagus nerves, although there are some differences between Eptatretid and Myxinid lineages (von Kupffer, 1900; Wicht and Northcutt, 1995; Braun and Northcutt, 1997; Ota and Kuratani, 2007; Baker et al., 2008). Molecular analyses have revealed that some cranial placodes in hagfishes express a suite of transcription factors and signaling molecules similar to that of both jawed vertebrates and lampreys, including expression of *Sox9* in the otic vesicle, and combined expression of *EbFgf8/17*, *EbSoxB1*, *EbPitxA*, and *EbLhx3/4A* in the nasohypophyseal region (Ota et al., 2007; Oisi et al., 2013b).

## Interaction of Neural Crest and Placodes in Cyclostomes

**Figure 4:**
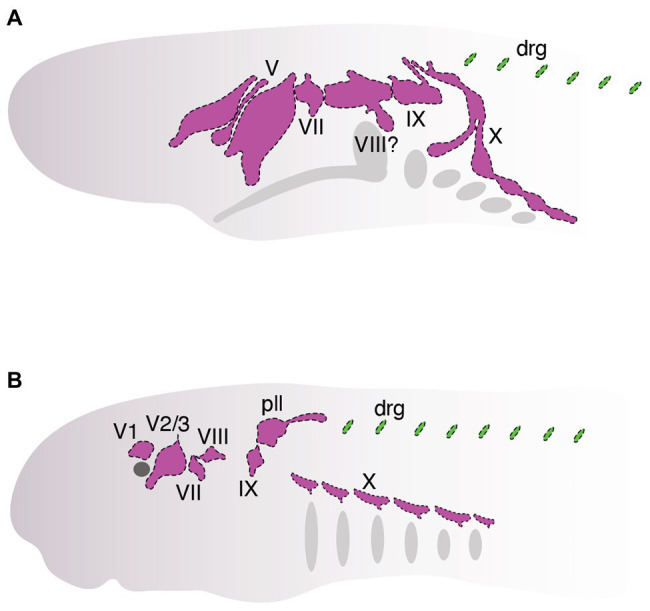
Organization of cranial sensory ganglia in hagfish (**A**, ~between stages. E. VIII and E. IX, modified from von Kupffer, 1900) and lamprey (**B**, Tahara stage 26, modified from Yuan et al., 2020). Cranial ganglia are colored pink and dorsal root ganglia are colored green. Ganglia are shown that can be homologized with those in jawed vertebrates (Figure 2).

Studies over the past several decades have found that neural crest cells and placodes in cyclostomes follow—with a few notable exceptions—a fairly typical course of development for vertebrates. Although we are now starting to get a deeper understanding of how neural crest and placodes develop in cyclostomes, it has remained unclear how these cells associate together within the embryonic cyclostome head to generate novel features such as cranial sensory ganglia (Figure 4) and how this compares to what we know about similar processes in jawed vertebrates. In this section, we discuss recent work that has begun to shed light on the matter specifically as it relates to the development of the cranial sensory ganglia in these animals (Figure 4). As noted above, in-depth cell lineage tracing and functional genetic analysis of neural crest and placodes is, for the most part, not feasible in hagfish. Consequently, almost all of our current understanding of neural crest and placode associations during cyclostome craniofacial development has come from data obtained from lampreys, given the relative ease with which their adults and embryos may be obtained and experimentally manipulated.

Cell lineage tracing by injection of vital lipophilic fluorescent dyes (DiI, DiO) has been a simple but powerful tool used in the lamprey embryological community for studying the origin, migration, and contributions of neural crest cells and placodes during development (Horigome et al., 1999; McCauley and Bronner-Fraser, 2003; Martin et al., 2009; Häming et al., 2011; Green et al., 2017). One early study by McCauley and Bronner-Fraser traced the contributions of neural crest cells to the lamprey head (McCauley and Bronner-Fraser, 2003). They found that DiI-labeled neural crest cells in the dorsal neural tube migrated in patterns similar to that of jawed vertebrates (McCauley and Bronner-Fraser, 2003). They observed cells migrating along dorsal-lateral and ventral pathways in the embryonic head and that colonized tissues that give rise to the oral and pharyngeal skeleton, as described in other vertebrates. The authors also revealed that lamprey cranial neural crest cells, particularly in the hindbrain, show very little restraint in their migration along the anterior-posterior axis, with cells often migrating far rostrally and/or caudally from their origin. Although the significance of this has remained unclear, one possibility is that hindbrain neural crest cells are free to colonize any one of the posterior pharyngeal arches because many of the cartilage elements in this region are almost identical along the anterior-posterior axis.

In addition to the head skeleton, which is almost entirely derived from cranial neural crest cells, it was found that neural crest cells also appeared to contribute to a subset of cranial sensory ganglia, which are derived uniquely from both neural crest and placodes. DiI-labeled neural crest cells were observed to colonize the ophthalmic and maxillomandibular lobes of the trigeminal ganglion and posterior lateral line ganglion (McCauley and Bronner-Fraser, 2003). Importantly, however, DiI experiments and immunostaining with a mouse *Sox10* antibody revealed that these neural crest cells seemingly surrounded—but were excluded from—the main core of each ganglion. This is unlike the condition in jawed vertebrates in which *Sox10*-positive neural crest cells colonize the core of several cranial sensory ganglia, where they give rise to cells of glial and/or neural origin. This peculiar feature of lamprey cranial ganglion development raised some important evolutionary questions. What is the functional role of neural crest cells in and their precise contributions to the development of a key vertebrate structure such as cranial sensory ganglia? How do these contributions compare to that of another vertebrate innovation, cranial placodes, and how do these cell populations interact together during head development to drive the formation of cranial sensory ganglia?

Partial answers to these questions have been provided recently by studies examining the roles of both neural crest and placodes during the development of cranial sensory ganglia in lampreys. Fate mapping of lamprey cranial ganglia by Modrell et al. (2014) has provided key insights into the relative contributions of neural crest and placode populations to these structures in jawless vertebrates (Modrell et al., 2014). Using a combination of immunohistochemistry and DiI labeling, Modrell et al. (2014) made some important observations. First, they found that placode-labeled cells in the ectoderm were internalized and eventually differentiated into neurons occupying the core of cranial ganglia, a result consistent with that described in jawed vertebrates. Second, they found that the cranial sensory ganglia of lampreys, unlike like that first described by McCauley and Bronner-Fraser, did indeed contain a complement of both neural crest and placodes, another result quite similar to that of jawed vertebrates. This discrepancy is likely related to the fact that the labeling experiments performed by Modrell et al. (2014) were done very early in development and they may have therefore labeled some of the early-delaminating placodes that could have been missed from earlier experiments. Somewhat surprisingly, however, the neural crest cells that colonized cranial ganglia never seemed to express genetic markers characteristic of differentiated neurons and were therefore considered to be of potential glial origin. Thus, unlike the case in jawed vertebrates, neural crest cells in lamprey did not seem to be a major contributor of sensory neurons to cranial ganglia, raising questions regarding the functional roles of the neural crest during cranial ganglion development in jawless vertebrates.

To address these issues, Yuan et al. (2020) combined *in situ* hybridization, immunohistochemistry, functional analysis by CRISPR/Cas9 genome editing, and two different fluorescent vital dyes to track the development of both neural crest (DiO) and placodes (DiI) simultaneously during lamprey development to: (1) identify how each cell population physically associated within cranial ganglia and (2) characterize the functional roles of neural crest and placodes during ganglion development. The results from this study were similar to those of Modrell et al. (2014) by demonstrating that cranial placodes were a major source of sensory neurons in the core of cranial ganglia. Another result shared between these two studies was the apparent absence of a prominent neuronal contribution by neural crest cells to the core of the cranial ganglia studied. Neural crest cells were observed to migrate and then surround and eventually envelop the core of placode-derived neurons in the ophthalmic and maxillomandibular lobes of the trigeminal ganglia, geniculate (facial) ganglion, and epibranchial (nodose) ganglia. These results were corroborated by gene expression analyses which revealed that the neural crest markers *TwistA* and *SoxE2* (Sauka-Spengler et al., 2007), rather than being expressed in the neuronal core of ganglia, were instead expressed in cells surrounding each ganglion. This situation is different from that of jawed vertebrates in which neural crest cells are a major source of sensory neurons within cranial ganglia. That said, it is important to emphasize that neither of the studies described here conclusively demonstrates a lack of any sensory neuron contributions of neural crest cells to cranial ganglia, given differences in the timing of dye labeling and the specific ganglia analyzed. At the very least, however, both studies did not identify a prominent role for cranial neural crest cells in this capacity in lamprey.

Finally, to tease apart the functional roles of both neural crest and placodes in lamprey cranial ganglion development, Yuan et al. (2020) used CRISPR/Cas9 genome editing to knock out the neural crest (*SoxE1* and *FoxD*-*A* mutants) and placodes (*DlxB* mutants) separately and then examined for defects in gangliogenesis. These experiments showed that genetic ablation of the neural crest did not impair the specification or migration of placodes. Rather, they found that all of the cranial sensory ganglia had abnormal morphologies, including inappropriate fusions of otherwise separate ganglia and, conversely, broken clusters of ganglia that are fused during normal development. Notably, none of the neural crest knockouts revealed any obvious loss of sensory neurons or ganglia. On the other hand, placode-specific knockouts (*DlxB*) consistently resulted in total or near-total loss of cranial ganglia (Yuan et al., 2020), although whether this effect is direct or indirect remains unknown. These results together suggest a patterning role for neural crest and a neurogenic role for placodes.

These recent studies have revealed some interesting similarities and differences regarding the developmental association of neural crest and placodes during lamprey craniofacial development relative to what has been described in jawed vertebrates. First, there is certainly evidence of deep evolutionary conservation regarding the overall developmental and genetic programs that guide the interaction of neural crest and placodes in the vertebrate head. For example, both lampreys and jawed vertebrates have more or less the same complement of cranial sensory ganglia, and both neural crest and placodes are each required for proper development of these structures. Additionally, there is evidence of evolutionary conservation of gene expression patterns in homologous placodes (e.g., *Six*, *Sox*, *Phox*, *Pax*, and *Dlx* expression in/around ganglia), with knockout experiments revealing that some of these genes are required for proper ganglion development across jawed and jawless vertebrate lineages (McCauley and Bronner-Fraser, 2002; Cerny et al., 2010; Modrell et al., 2014; Yuan et al., 2020).

In contrast to evolutionary conservation, there are also clear differences in the developmental associations of neural crest and placodes in the heads of jawed and jawless vertebrates. Most notably, a prominent neural crest contribution of sensory neurons to the core of cranial ganglia is conspicuously absent during stages of lamprey development that have been examined. Instead, cranial sensory neurons in the lamprey head seem to be derived almost entirely from placodes. Although there is marked variation in the extent to which neural crest cells do (maxillomandibular trigeminal) or do not (ophthalmic trigeminal) contribute sensory neurons to cranial ganglia in jawed vertebrates, lampreys are the only vertebrates, to our knowledge, that appear to lack any neural crest contribution of sensory neurons in cranial ganglia (Modrell et al., 2014; Yuan et al., 2020). Instead, cranial neural crest cells in lampreys may play important functional roles in cranial ganglion morphogenesis, in which placodes condense into clusters of differentiated neurons that become enveloped by migratory cranial neural crest cells to shape the functional morphology of these neuronal clusters. A similar role has also been described in jawed vertebrates in which neural crest cells form “corridors” that guide and physically shape placode-derived sensory neurons in the head (Freter et al., 2013). The molecular mechanisms of these types of interactions in jawed vertebrates seem to involve intercellular signaling pathways and adhesion proteins (Shiau et al., 2008; Wu et al., 2014; Shah and Taneyhill, 2015; Wu and Taneyhill, 2019), which have been shown recently to influence the patterning of cranial ganglia in lampreys (York et al., 2018).

**Figure 5:**
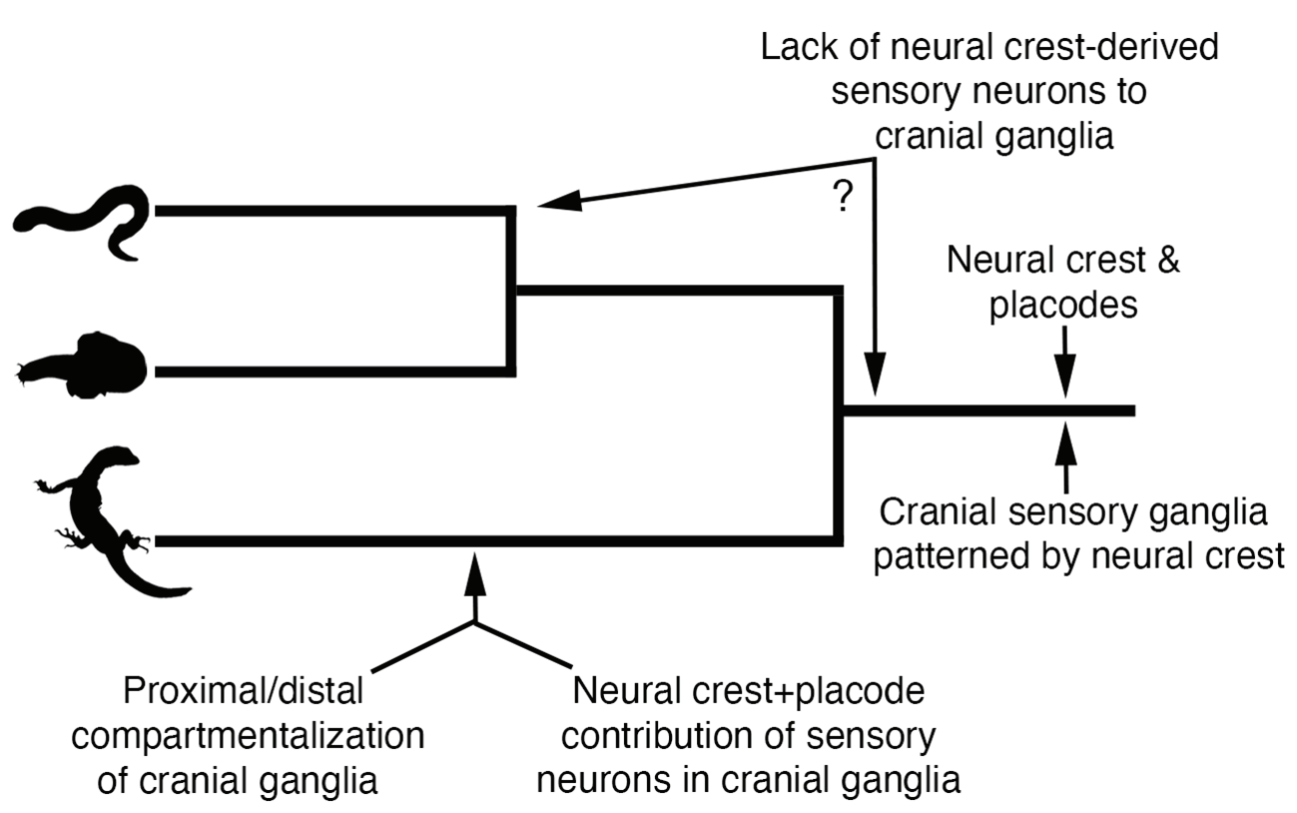
Model for evolution of neural crest and placode associations during craniofacial development in vertebrates. Phylogenetic relationships are depicted for lampreys **(top panel)**, hagfishes **(middle panel)**, and jawed vertebrates **(bottom panel)**.

By comparing neural crest and placode development across jawed and jawless vertebrates, we can begin to make some inferences regarding how these cell populations may have become associated in ancestral vertebrates to coordinate the development of key craniofacial structures (Figure 5). First, we can be fairly certain that early jawless vertebrates had both neural crest cells and placodes. Second, we can also be confident that the developmental mechanisms of each cell population were likely similar to that of extant vertebrates. Third, ancestral vertebrates possessed cranial sensory ganglia that were likely shaped by the developmental coordination of both neural crest and placodes. Finally, comparative embryology studies have shown no evidence that the cranial sensory ganglia of jawless vertebrates are compartmentalized into the morphologically or functionally distinct neural crest-derived (proximal) and placode-derived (distal) components that is characteristic of gnathostomes (Kuratani et al., 1997; Modrell et al., 2014; Pombal and Megías, 2019).

The recent work on neural crest and placode interactions in lamprey embryos compared with our current understanding of this process in jawed vertebrates, allows us to infer what might have been the ancestral vertebrate condition. One possibility is that cranial neural crest cells in the first vertebrates would have likely played a very minor role, if any, in the contribution of sensory neurons to the core of cranial ganglia, a role fulfilled instead by neurogenic placodes. Rather, cranial neural crest cells in ancestral vertebrates would have been important in patterning and shaping the morphology of the placode-derived sensory neurons occupying the core of cranial ganglia, with this feature still being retained in jawed vertebrates. This model suggests that the functional roles of neural crest and placodes in early jawless vertebrates were distinct and that the dual neural crest and placode origin of sensory neurons in cranial ganglia would have likely evolved along stem lineages leading to crown group jawed vertebrates (Figure 5). Thus, the overall trend in vertebrate evolution would have been the gradual mixing and integration of sensory neurons of both placode and neural crest origin, with a subset of ganglia (e.g., VII, IX, and X) incorporating neural crest and placodes into distinct proximal and distal aspects, respectively. It is important to point out that this scenario takes into account only information available from one lineage of extant jawless vertebrates, the lampreys. Detailed analysis of neural crest and placode contributions—and their interactions—in hagfish will be important for determining the polarity of character evolution and to better understand the roles of neural crest and placode interactions in the evolution of vertebrate craniofacial development.

## Author Contributions

JY wrote the manuscript. DM, TY, and JY edited the manuscript. All authors contributed to the article and approved the submitted version.

### Conflict of Interest

The authors declare that the research was conducted in the absence of any commercial or financial relationships that could be construed as a potential conflict of interest.

## References

[ref1] Baker C. V. H. Bronner-Fraser M. (1997). The origins of the neural crest. Part I: embryonic induction. Mech. Dev. 69, 3–11. 10.1016/s0925-4773(97)00132-9, PMID: 9486527

[ref2] Baker C. V. H. Bronner-Fraser M. (2000). Establishing neuronal identity in vertebrate neurogenic placodes. Development 127, 3045–3056. PMID: 10862742 10.1242/dev.127.14.3045

[ref3] Baker C. V. H. Bronner-Fraser M. (2001). Vertebrate cranial placodes I. embryonic induction. Dev. Biol. 232, 1–61. 10.1006/dbio.2001.0156, PMID: 11254347

[ref4] Baker C. V. H. O’neill P. Mccole R. B. (2008). Lateral line, otic and epibranchial placodes: developmental and evolutionary links? J. Exp. Zool. B Mol. Dev. Evol. 310, 370–383. 10.1002/jez.b.21188, PMID: 17638322 PMC4209393

[ref5] Barlow L. A. (2002). Cranial nerve development: placodal neurons ride the crest. Curr. Biol. 12, R171–R173. 10.1016/S0960-9822(02)00734-0, PMID: 11882306

[ref6] Begbie J. Graham A. (2001). Integration between the epibranchial placodes and the hindbrain. Science 294, 595–598. 10.1126/science.1062028, PMID: 11641498

[ref7] Braun C. B. Northcutt R. G. (1997). The lateral line system of hagfishes (Craniata: Myxinoidea). Acta Zool. 78, 247–268. 10.1111/j.1463-6395.1997.tb01010.x

[ref8] Brazeau M. D. Friedman M. (2014). The characters of Palaeozoic jawed vertebrates. Zool. J. Linnean Soc. 170, 779–821. 10.1111/zoj.12111, PMID: 25750460 PMC4347021

[ref9] Brazeau M. D. Friedman M. (2015). The origin and early phylogenetic history of jawed vertebrates. Nature 520, 490–497. 10.1038/nature14438, PMID: 25903631 PMC4648279

[ref10] Bronner M. (2014). Migrating into genomics with the neural crest. Adv. Biol. 2014, 1–8. 10.1155/2014/264069

[ref11] Bullock T. H. Moore J. K. Fields R. D. (1984). Evolution of myelin sheaths: both lamprey and hagfish lack myelin. Neurosci. Lett. 48, 145–148. 10.1016/0304-3940(84)90010-7, PMID: 6483278

[ref12] Cattell M. Lai S. Cerny R. Medeiros D. M. (2011). A new mechanistic scenario for the origin and evolution of vertebrate cartilage. PLoS One 6:e22474. 10.1371/journal.pone.0022474, PMID: 21799866 PMC3142159

[ref13] Cerny R. Cattell M. Sauka-Spengler T. Bronner-Fraser M. Yu F. Q. Medeiros D. M. (2010). Evidence for the prepattern/cooption model of vertebrate jaw evolution. Proc. Natl. Acad. Sci. U. S. A. 107, 17262–17267. 10.1073/pnas.1009304107, PMID: 20855630 PMC2951391

[ref14] Cheung M. Tai A. Lu P. J. Cheah K. S. (2019). Acquisition of multipotent and migratory neural crest cells in vertebrate evolution. Curr. Opin. Genet. Dev. 57, 84–90. 10.1016/j.gde.2019.07.018, PMID: 31470291

[ref15] Conel J. L. (1942). The origin of the neural crest. J. Comp. Neurol. 76, 191–215. 10.1002/cne.900760202

[ref16] Couly G. F. Coltey P. M. Le Douarin N. M. (1993). The triple origin of skull in higher vertebrates: a study in quail-chick chimeras. Development 117, 409–429. PMID: 8330517 10.1242/dev.117.2.409

[ref17] Dahl E. Koseki H. Balling R. (1997). Pax genes and organogenesis. BioEssays 19, 755–765. 10.1002/bies.950190905, PMID: 9297966

[ref18] Damas H. (1943). Recherches sur le développement de Lampetra fluviatilis L.: contribution à l'étude de la Céphalogenèse des Vertébrés. Paris: H. Vaillant-Carmanne.

[ref19] D’amico-Martel A. Noden D. M. (1983). Contributions of placodal and neural crest cells to avian cranial peripheral ganglia. Am. J. Anat. 166, 445–468. 10.1002/aja.1001660406, PMID: 6858941

[ref20] Fish J. L. (2019). Evolvability of the vertebrate craniofacial skeleton. Semin. Cell Dev. Biol. 91, 13–22. 10.1016/j.semcdb.2017.12.004, PMID: 29248471 PMC5999547

[ref21] Freter S. Fleenor S. J. Freter R. Liu K. J. Begbie J. (2013). Cranial neural crest cells form corridors prefiguring sensory neuroblast migration. Development 140, 3595–3600. 10.1242/dev.091033, PMID: 23942515 PMC3742142

[ref22] Froriep A. (1885). Ueber Anlagen von Sinnesorganen am Facialis, Glossopharyngeus und Vagus, über die genetische Stellung des Vagus zum Hypoglossus, und über die Herkunft der Zungenmuskulatur: Beitrag zur Entwickelungsgeschichte des Säugethierkopfes. Leipzig: éditeur inconnu.

[ref23] Gans C. Northcutt R. G. (1983). Neural crest and the origin of vertebrates: a new head. Science 220, 268–274. 10.1126/science.220.4594.268, PMID: 17732898

[ref24] Gassmann M. Casagranda F. Orioli D. Simon H. Lai C. Klein R.. (1995). Aberrant neural and cardiac development in mice lacking the ErbB4 neuregulin receptor. Nature 378, 390–394. 10.1038/378390a0, PMID: 7477376

[ref25] Gillis J. A. Alsema E. C. Criswell K. E. (2017). Trunk neural crest origin of dermal denticles in a cartilaginous fish. Proc. Natl. Acad. Sci. U. S. A. 114, 13200–13205. 10.1073/pnas.1713827114, PMID: 29158384 PMC5740611

[ref26] Goldberg S. Venkatesh A. Martinez J. Dombroski C. Abesamis J. Campbell C.. (2020). The development of the trunk neural crest in the turtle *Trachemys scripta*. Dev. Dyn. 249, 125–140. 10.1002/dvdy.119, PMID: 31587387 PMC7293771

[ref27] Golding J. P. Sobieszczuk D. Dixon M. Coles E. Christiansen J. Wilkinson D.. (2004). Roles of erbB4, rhombomere-specific, and rhombomere-independent cues in maintaining neural crest-free zones in the embryonic head. Dev. Biol. 266, 361–372. 10.1016/j.ydbio.2003.11.003, PMID: 14738883

[ref28] Green S. A. Bronner M. E. (2014). The lamprey: a jawless vertebrate model system for examining origin of the neural crest and other vertebrate traits. Differentiation 87, 44–51. 10.1016/j.diff.2014.02.001, PMID: 24560767 PMC3995830

[ref29] Green S. A. Simões-Costa M. Bronner M. E. (2015). Evolution of vertebrates as viewed from the crest. Nature 520, 474–482. 10.1038/nature14436, PMID: 25903629 PMC5100666

[ref30] Green S. A. Uy B. R. Bronner M. E. (2017). Ancient evolutionary origin of vertebrate enteric neurons from trunk-derived neural crest. Nature 544, 88–91. 10.1038/nature21679, PMID: 28321127 PMC5383518

[ref31] Grocott T. Tambalo M. Streit A. (2012). The peripheral sensory nervous system in the vertebrate head: a gene regulatory perspective. Dev. Biol. 370, 3–23. 10.1016/j.ydbio.2012.06.028, PMID: 22790010

[ref32] Hall B. K. (2008). The neural crest and neural crest cells in vertebrate development and evolution. New York: Springer.

[ref33] Hall B. K. (2018). Germ layers, the neural crest and emergent organization in development and evolution. Genesis 56:e23103. 10.1002/dvg.23103, PMID: 29637683

[ref34] Hamburger V. (1961). Experimental analysis of the dual origin of the trigeminal ganglion in the chick embryo. J. Exp. Zool. 148, 91–123. 10.1002/jez.1401480202, PMID: 13904079

[ref35] Häming D. Simões-Costa M. Uy B. Valencia J. Sauka-Spengler T. Bronner-Fraser M. (2011). Expression of sympathetic nervous system genes in lamprey suggests their recruitment for specification of a new vertebrate feature. PLoS One 6:e26543. 10.1371/journal.pone.0026543, PMID: 22046306 PMC3203141

[ref36] Hardisty M. W. (1979). Biology of the cyclostomes. USA: Springer.

[ref37] Heimberg A. M. Cowper-Sal-Lari R. Sémon M. Donoghue P. C. J. Peterson K. J. (2010). microRNAs reveal the interrelationships of hagfish, lampreys, and gnathostomes and the nature of the ancestral vertebrate. Proc. Natl. Acad. Sci. U. S. A. 107, 19379–19383. 10.1073/pnas.1010350107, PMID: 20959416 PMC2984222

[ref38] His W. (1868). Untersuchungen über die erste Anlage des Wirbelthierleibes: die erste Entwickelung des Hühnchens im Ei. Leipzig: FCW Vogel.

[ref39] Hockman D. Burns A. J. Schlosser G. Gates K. P. Jevans B. Mongera A.. (2017). Evolution of the hypoxia-sensitive cells involved in amniote respiratory reflexes. Elife 6:e21231. 10.7554/eLife.21231, PMID: 28387645 PMC5438250

[ref40] Hockman D. Chong-Morrison V. Green S. A. Gavriouchkina D. Candido-Ferreira I. Ling I. T.. (2019). A genome-wide assessment of the ancestral neural crest gene regulatory network. Nat. Commun. 10:4689. 10.1038/s41467-019-12687-4, PMID: 31619682 PMC6795873

[ref41] Holland N. D. (2007). Hagfish embryos again—the end of a long drought. BioEssays 29, 833–836. 10.1002/bies.20620, PMID: 17691082

[ref42] Horie R. Hazbun A. Chen K. Cao C. Levine M. Horie T. (2018). Shared evolutionary origin of vertebrate neural crest and cranial placodes. Nature 560, 228–232. 10.1038/s41586-018-0385-7, PMID: 30069052 PMC6390964

[ref43] Horigome N. Myojin M. Ueki T. Hirano S. Aizawa S. Kuratani S. (1999). Development of cephalic neural crest cells in embryos of *Lampetra japonica*, with special reference to the evolution of the jaw. Dev. Biol. 207, 287–308. 10.1006/dbio.1998.9175, PMID: 10068464

[ref44] Jandzik D. Hawkins M. B. Cattell M. V. Cerny R. Square T. A. Medeiros D. M. (2014). Roles for FGF in lamprey pharyngeal pouch formation and skeletogenesis highlight ancestral functions in the vertebrate head. Development 141, 629–638. 10.1242/dev.097261, PMID: 24449839

[ref45] Kuraku S. Takio Y. Sugahara F. Takechi M. Kuratani S. (2010). Evolution of oropharyngeal patterning mechanisms involving dlx and endothelins in vertebrates. Dev. Biol. 341, 315–323. 10.1016/j.ydbio.2010.02.013, PMID: 20171204

[ref46] Kuratani S. (2008). Is the vertebrate head segmented?—evolutionary and developmental considerations. Integr. Comp. Biol. 48, 647–657. 10.1093/icb/icn015, PMID: 20607133 PMC2895337

[ref47] Kuratani S. Ahlberg P. E. (2018). Evolution of the vertebrate neurocranium: problems of the premandibular domain and the origin of the trabecula. Zool. Lett. 4:1. 10.1186/s40851-017-0083-6, PMID: 29340168 PMC5759263

[ref48] Kuratani S. C. Miyagawa-Tomita S. Kirby M. L. (1991). Development of cranial nerves in the chick embryo with special reference to the alterations of cardiac branches after ablation of the cardiac neural crest. Anat. Embryol. 183, 501–514. 10.1007/BF00186439, PMID: 1862951

[ref49] Kuratani S. Ota K. G. (2008). Hagfish (Cyclostomata, vertebrata): searching for the ancestral developmental plan of vertebrates. BioEssays 30, 167–172. 10.1002/bies.20701, PMID: 18197595

[ref50] Kuratani S. Ueki T. Aizawa S. Hirano S. (1997). Peripheral development of cranial nerves in a cyclostome, *Lampetra japonica*: morphological distribution of nerve branches and the vertebrate body plan. J. Comp. Neurol. 384, 483–500. 10.1002/(SICI)1096-9861(19970811)384:4<483::AID-CNE1>3.0.CO;2-Z, PMID: 9259485

[ref51] Lakiza O. Miller S. Bunce A. Lee E. M. J. Mccauley D. W. (2011). SoxE gene duplication and development of the lamprey branchial skeleton: insights into development and evolution of the neural crest. Dev. Biol. 359, 149–161. 10.1016/j.ydbio.2011.08.012, PMID: 21889937

[ref52] Langille R. M. Hall B. K. (1988). Role of the neural crest in development of the trabeculae and branchial arches in embryonic sea lamprey, *Petromyzon marinus*. Development 102, 301–310.

[ref53] Lassiter R. N. Stark M. R. Zhao T. Zhou C. J. (2014). Signaling mechanisms controlling cranial placode neurogenesis and delamination. Dev. Biol. 389, 39–49. 10.1016/j.ydbio.2013.11.025, PMID: 24315854 PMC3972360

[ref54] Le Douarin N. Kalcheim C. (1999). The neural crest. New York: Cambridge University Press.

[ref55] Li Y. Vieceli F. M. Gonzalez W. G. Li A. Tang W. Lois C.. (2019). In vivo quantitative imaging provides insights into trunk neural crest migration. Cell Rep. 26, 1489.e1483–1500.e1483. 10.1016/j.celrep.2019.01.039, PMID: 30726733 PMC6449054

[ref56] Ling I. T. Sauka-Spengler T. (2019). Early chromatin shaping predetermines multipotent vagal neural crest into neural, neuronal and mesenchymal lineages. Nat. Cell Biol. 21, 1504–1517. 10.1038/s41556-019-0428-9, PMID: 31792380 PMC7188519

[ref57] Lukoseviciute M. Gavriouchkina D. Williams R. M. Hochgreb-Hagele T. Senanayake U. Chong-Morrison V.. (2018). From pioneer to repressor: bimodal foxd3 activity dynamically remodels neural crest regulatory landscape in vivo. Dev. Cell 47, 608.e606–628.e606. 10.1016/j.devcel.2018.11.009, PMID: 30513303 PMC6286384

[ref58] Lwigale P. Y. (2001). Embryonic origin of avian corneal sensory nerves. Dev. Biol. 239, 323–337. 10.1006/dbio.2001.0450, PMID: 11784038

[ref59] Maharana S. K. Riddiford N. Schlosser G. (2017). Gene regulatory networks for cranial placode development up-and downstream of Six1 and Eya1. Mech. Dev. 145:S140. 10.1016/j.mod.2017.04.393

[ref60] Maharana S. K. Schlosser G. (2018). A gene regulatory network underlying the formation of pre-placodal ectoderm in *Xenopus laevis*. BMC Biol. 16:79. 10.1186/s12915-018-0540-5, PMID: 30012125 PMC6048776

[ref61] Mansouri A. Hallonet M. Gruss P. (1996). Pax genes and their roles in cell differentiation and development. Curr. Opin. Cell Biol. 8, 851–857. 10.1016/S0955-0674(96)80087-1, PMID: 8939674

[ref62] Martik M. L. Bronner M. E. (2017). Regulatory logic underlying diversification of the neural crest. Trends Genet. 33, 715–727. 10.1016/j.tig.2017.07.015, PMID: 28851604 PMC5610108

[ref63] Martik M. L. Gandhi S. Uy B. R. Gillis J. A. Green S. A. Simoes-Costa M.. (2019). Evolution of the new head by gradual acquisition of neural crest regulatory circuits. Nature 574, 675–678. 10.1038/s41586-019-1691-4, PMID: 31645763 PMC6858584

[ref64] Martin W. M. Bumm L. A. Mccauley D. W. (2009). Development of the viscerocranial skeleton during embryogenesis of the sea lamprey, *Petromyzon marinus*. Dev. Dyn. 238, 3126–3138. 10.1002/dvdy.22164, PMID: 19924811

[ref65] McCauley D. W. Bronner-Fraser M. (2002). Conservation of Pax gene expression in ectodermal placodes of the lamprey. Gene 287, 129–139. 10.1016/S0378-1119(01)00894-0, PMID: 11992731

[ref66] McCauley D. W. Bronner-Fraser M. (2003). Neural crest contributions to the lamprey head. Development 130, 2317–2327. 10.1242/dev.00451, PMID: 12702647

[ref67] McCauley D. W. Bronner-Fraser M. (2006). Importance of SoxE in neural crest development and the evolution of the pharynx. Nature 441, 750–752. 10.1038/nature04691, PMID: 16760978

[ref68] McCauley D. W. Docker M. F. Whyard S. Li W. (2015). Lampreys as diverse model organisms in the genomics era. Bioscience 65, 1046–1056. 10.1093/biosci/biv139, PMID: 26951616 PMC4777059

[ref69] Meulemans D. Bronner-Fraser M. (2002). Amphioxus and lamprey AP-2 genes: implications for neural crest evolution and migration patterns. Development 129, 4953–4962. PMID: 12397104 10.1242/dev.129.21.4953

[ref70] Meulemans D. Bronner-Fraser M. (2004). Gene-regulatory interactions in neural crest evolution and development. Dev. Cell 7, 291–299. 10.1016/j.devcel.2004.08.007, PMID: 15363405

[ref71] Meulemans D. Mccauley D. Bronner-Fraser M. (2003). Id expression in amphioxus and lamprey highlights the role of gene cooption during neural crest evolution. Dev. Biol. 264, 430–442. 10.1016/j.ydbio.2003.09.006, PMID: 14651928

[ref72] Milet C. Monsoro-Burq A. H. (2012). Neural crest induction at the neural plate border in vertebrates. Dev. Biol. 366, 22–33. 10.1016/j.ydbio.2012.01.013, PMID: 22305800

[ref73] Miyashita T. Coates M. I. Farrar R. Larson P. Manning P. L. Wogelius R. A.. (2019). Hagfish from the cretaceous Tethys Sea and a reconciliation of the morphological—molecular conflict in early vertebrate phylogeny. Proc. Natl. Acad. Sci. U. S. A. 116, 2146–2151. 10.1073/pnas.1814794116, PMID: 30670644 PMC6369785

[ref74] Modrell M. S. Hockman D. Uy B. Buckley D. Sauka-Spengler T. Bronner M.. (2014). A fate-map for cranial sensory ganglia in the sea lamprey. Dev. Biol. 385, 405–416. 10.1016/j.ydbio.2013.10.021, PMID: 24513489 PMC3928997

[ref75] Moody S. A. Lamantia A. -S. (2015). Transcriptional regulation of cranial sensory placode development. Curr. Top. Dev. Biol. 111, 301–350. 10.1016/bs.ctdb.2014.11.009, PMID: 25662264 PMC4425424

[ref76] Murakami Y. Ogasawara M. Sugahara F. Hirano S. Satoh N. Kuratani S. (2001). Identification and expression of the lamprey Pax6 gene: evolutionary origin of the segmented brain of vertebrates. Development 128, 3521–3531. PMID: 11566857 10.1242/dev.128.18.3521

[ref77] Nelson J. S. Grande T. C. Wilson M. V. (2016). Fishes of the world. Hoboken, New Jersey: John Wiley and Sons.

[ref78] Newth D. R. (1950). Fate of the neural crest in lampreys. Nature 165, 284–284. 10.1038/165284a0, PMID: 15405801

[ref79] Newth D. R. (1951). Experiments on the neural crest of the lamprey embryo. J. Exp. Biol. 28, 247–260.

[ref80] Newth D. R. (1956). On the neural crest of the lamprey embryo. J. Embryol. Exp. Morpholog. 4, 358–375.

[ref81] Nikitina N. Sauka-Spengler T. Bronner-Fraser M. (2008). Dissecting early regulatory relationships in the lamprey neural crest gene network. Proc. Natl. Acad. Sci. U. S. A. 105, 20083–20088. 10.1073/pnas.0806009105, PMID: 19104059 PMC2629288

[ref82] Nikitina N. Tong L. Bronner M. (2011). Ancestral network module regulating prdm1 expression in the lamprey neural plate border. Dev. Dyn. 240, 2265–2271. 10.1002/dvdy.22720, PMID: 21932309 PMC3277493

[ref83] Northcutt R. G. (2005). The new head hypothesis revisited. J. Exp. Zool. B Mol. Dev. Evol. 304B, 274–297. 10.1002/jez.b.21063, PMID: 16003768

[ref84] Northcutt R. G. Gans C. (1983). The genesis of neural crest and epidermal placodes: a reinterpretation of vertebrate origins. Q. Rev. Biol. 58, 1–28. 10.1086/413055, PMID: 6346380

[ref85] Nyut D. R. (1955). The neural crest and the head skeleton of lampreys. Dokl. Akad. Nauk SSSR 102, 653–656. PMID: 13241357

[ref86] Oisi Y. Ota K. G. Fujimoto S. Kuratani S. (2013a). Development of the chondrocranium in hagfishes, with special reference to the early evolution of vertebrates. Zool. Sci. 30, 944–961. 10.2108/zsj.30.944, PMID: 24199860

[ref87] Oisi Y. Ota K. G. Kuraku S. Fujimoto S. Kuratani S. (2013b). Craniofacial development of hagfishes and the evolution of vertebrates. Nature 493, 175–180. 10.1038/nature11794 23254938

[ref88] Osborne N. J. Begbie J. Chilton J. K. Schmidt H. Eickholt B. J. (2005). Semaphorin/neuropilin signaling influences the positioning of migratory neural crest cells within the hindbrain region of the chick. Dev. Dyn. 232, 939–949. 10.1002/dvdy.20258, PMID: 15729704

[ref89] Ota K. G. Kuraku S. Kuratani S. (2007). Hagfish embryology with reference to the evolution of the neural crest. Nature 446, 672–675. 10.1038/nature05633, PMID: 17377535

[ref90] Ota K. G. Kuratani S. (2007). Cyclostome embryology and early evolutionary history of vertebrates. Integr. Comp. Biol. 47, 329–337. 10.1093/icb/icm022, PMID: 21672842

[ref91] Ota K. G. Kuratani S. (2008). Developmental biology of hagfishes, with a report on newly obtained embryos of the Japanese inshore hagfish, *Eptatretus burgeri*. Zool. Sci. 25, 999–1011. 10.2108/zsj.25.999, PMID: 19267636

[ref92] Parker H. J. De Kumar B. Green S. A. Prummel K. D. Hess C. Kaufman C. K.. (2019). A Hox-TALE regulatory circuit for neural crest patterning is conserved across vertebrates. Nat. Commun. 10:1189. 10.1038/s41467-019-09197-8, PMID: 30867425 PMC6416258

[ref93] Parker H. J. Sauka-Spengler T. Bronner M. Elgar G. (2014). A reporter assay in lamprey embryos reveals both functional conservation and elaboration of vertebrate enhancers. PLoS One 9:e85492. 10.1371/journal.pone.0085492, PMID: 24416417 PMC3887057

[ref94] Patthey C. Schlosser G. Shimeld S. M. (2014). The evolutionary history of vertebrate cranial placodes–I: cell type evolution. Dev. Biol. 389, 82–97. 10.1016/j.ydbio.2014.01.017, PMID: 24495912

[ref95] Piotrowski T. Baker C. V. (2014). The development of lateral line placodes: taking a broader view. Dev. Biol. 389, 68–81. 10.1016/j.ydbio.2014.02.016, PMID: 24582732

[ref96] Pla P. Monsoro-Burq A. H. (2018). The neural border: induction, specification and maturation of the territory that generates neural crest cells. Dev. Biol. 444, S36–S46. 10.1016/j.ydbio.2018.05.018, PMID: 29852131

[ref97] Platt J. B. (1894). Ectodermic origin of the cartilages of the head. Anat. Anz. 1893, 506–509.

[ref98] Plouhinec J. -L. Medina-Ruiz S. Borday C. Bernard E. Vert J. -P. Eisen M. B.. (2017). A molecular atlas of the developing ectoderm defines neural, neural crest, placode, and nonneural progenitor identity in vertebrates. PLoS Biol. 15:e2004045. 10.1371/journal.pbio.2004045, PMID: 29049289 PMC5663519

[ref99] Pombal M. A. Megías M. (2019). Development and functional organization of the cranial nerves in lampreys. Anat. Rec. 302, 512–539. 10.1002/ar.23821, PMID: 29659164

[ref100] Riddiford N. Schlosser G. (2016). Dissecting the pre-placodal transcriptome to reveal presumptive direct targets of Six1 and Eya1 in cranial placodes. Elife 5:e17666. 10.7554/eLife.17666, PMID: 27576864 PMC5035141

[ref101] Rothstein M. Simões-Costa M. (2020). Heterodimerization of TFAP2 pioneer factors drives epigenomic remodeling during neural crest specification. Genome Res. 30, 35–48. 10.1101/gr.249680.119, PMID: 31848212 PMC6961570

[ref102] Saint-Jeannet J. -P. Moody S. A. (2014). Establishing the pre-placodal region and breaking it into placodes with distinct identities. Dev. Biol. 389, 13–27. 10.1016/j.ydbio.2014.02.011, PMID: 24576539 PMC3985045

[ref103] Santagati F. Rijli F. M. (2003). Cranial neural crest and the building of the vertebrate head. Nat. Rev. Neurosci. 4, 806–818. 10.1038/nrn1221, PMID: 14523380

[ref104] Sauka-Spengler T. Bronner-Fraser M. (2008). Insights from a sea lamprey into the evolution of neural crest gene regulatory network. Biol. Bull. 214, 303–314. 10.2307/25470671, PMID: 18574106

[ref105] Sauka-Spengler T. Meulemans D. M. Jones M. Bronner-Fraser M. (2007). Ancient evolutionary origin of the neural crest gene regulatory network. Dev. Cell 13, 405–420. 10.1016/j.devcel.2007.08.005, PMID: 17765683

[ref106] Schlosser G. (2002). Development and evolution of lateral line placodes in amphibians I. Development. Zoology 105, 119–146. 10.1078/0944-2006-00058, PMID: 16351862

[ref107] Schlosser G. (2005). Evolutionary origins of vertebrate placodes: insights from developmental studies and from comparisons with other deuterostomes. J. Exp. Zool. B Mol. Dev. Evol. 304, 347–399. 10.1002/jez.b.21055, PMID: 16003766

[ref108] Schlosser G. (2006). Induction and specification of cranial placodes. Dev. Biol. 294, 303–351. 10.1016/j.ydbio.2006.03.009, PMID: 16677629

[ref109] Schlosser G. (2010). “Making senses: development of vertebrate cranial placodes” in International review of cell and molecular biology. ed. Jeon K. (Cambridge: Elsevier), 129–234. 10.1016/S1937-6448(10)83004-7 20801420

[ref110] Schlosser G. (2017). From so simple a beginning–what amphioxus can teach us about placode evolution. Int. J. Dev. Biol. 61, 633–648. 10.1387/ijdb.170127gs, PMID: 29319112

[ref111] Schlosser G. Ahrens K. (2004). Molecular anatomy of placode development in *Xenopus laevis*. Dev. Biol. 271, 439–466. 10.1016/j.ydbio.2004.04.013, PMID: 15223346

[ref112] Schlosser G. Northcutt R. G. (2000). Development of neurogenic placodes in *Xenopus laevis*. J. Comp. Neurol. 418, 121–146. 10.1002/(SICI)1096-9861(20000306)418:2<121::AID-CNE1>3.0.CO;2-M, PMID: 10701439

[ref113] Schwarz Q. Vieira J. M. Howard B. Eickholt B. J. Ruhrberg C. (2008). Neuropilin 1 and 2 control cranial gangliogenesis and axon guidance through neural crest cells. Development 135, 1605–1613. 10.1242/dev.015412, PMID: 18356247 PMC2705499

[ref114] Shah A. Taneyhill L. A. (2015). Differential expression pattern of Annexin A6 in chick neural crest and placode cells during cranial gangliogenesis. Gene Expr. Patterns 18, 21–28. 10.1016/j.gep.2015.05.001, PMID: 25976293 PMC4516561

[ref115] Shiau C. E. Lwigale P. Y. Das R. M. Wilson S. A. Bronner-Fraser M. (2008). Robo2-Slit1 dependent cell-cell interactions mediate assembly of the trigeminal ganglion. Nat. Neurosci. 11, 269–276. 10.1038/nn2051, PMID: 18278043

[ref116] Shigetani Y. Sugahara F. Kawakami Y. Murakami Y. Hirano S. Kuratani S. (2002). Heterotopic shift of epithelial-mesenchymal interactions in vertebrate jaw evolution. Science 296, 1316–1319. 10.1126/science.1068310, PMID: 12016315

[ref117] Shimeld S. M. Donoghue P. C. J. (2012). Evolutionary crossroads in developmental biology: cyclostomes (lamprey and hagfish). Development 139, 2091–2099. 10.1242/dev.074716, PMID: 22619386

[ref118] Simões-Costa M. Bronner M. E. (2015). Establishing neural crest identity: a gene regulatory recipe. Development 142, 242–257. 10.1242/dev.105445, PMID: 25564621 PMC4302844

[ref119] Singh S. Groves A. K. (2016). The molecular basis of craniofacial placode development. Wiley Interdiscip. Rev. Dev. Biol. 5, 363–376. 10.1002/wdev.226, PMID: 26952139 PMC4833591

[ref120] Smith J. J. Kuraku S. Holt C. Sauka-Spengler T. Jiang N. Campbell M. S.. (2013). Sequencing of the sea lamprey (*Petromyzon marinus*) genome provides insights into vertebrate evolution. Nat. Genet. 45, 415–421. 10.1038/ng.2568, PMID: 23435085 PMC3709584

[ref121] Smith J. J. Timoshevskaya N. Ye C. Holt C. Keinath M. C. Parker H. J.. (2018). The sea lamprey germline genome provides insights into programmed genome rearrangement and vertebrate evolution. Nat. Genet. 50, 270–277. 10.1038/s41588-017-0036-1, PMID: 29358652 PMC5805609

[ref122] Soldatov R. Kaucka M. Kastriti M. E. Petersen J. Chontorotzea T. Englmaier L.. (2019). Spatiotemporal structure of cell fate decisions in murine neural crest. Science 364:eaas9536. 10.1126/science.aas9536, PMID: 31171666

[ref123] Square T. Jandzik D. Cattell M. Hansen A. Medeiros D. M. (2016a). Embryonic expression of endothelins and their receptors in lamprey and frog reveals stem vertebrate origins of complex Endothelin signaling. Sci. Rep. 6:34282. 10.1038/srep34282, PMID: 27677704 PMC5039696

[ref124] Square T. Jandzik D. Romášek M. Cerny R. Medeiros D. (2016b). The origin and diversification of the developmental mechanisms that pattern the vertebrate head skeleton. Dev. Biol. 427, 219–229. 10.1016/j.ydbio.2016.11.014, PMID: 27884657

[ref125] Square T. Romasek M. Jandzik D. Cattell M. V. Klymkowsky M. Medeiros D. M. (2015). CRISPR/Cas9-mediated mutagenesis in the sea lamprey *Petromyzon marinus*: a powerful tool for understanding ancestral gene functions in vertebrates. Development 142, 4180–4187. 10.1242/dev.125609, PMID: 26511928 PMC4712834

[ref126] Steventon B. Mayor R. Streit A. (2014). Neural crest and placode interaction during the development of the cranial sensory system. Dev. Biol. 389, 28–38. 10.1016/j.ydbio.2014.01.021, PMID: 24491819 PMC4439187

[ref127] Streit A. (2018). Specification of sensory placode progenitors: signals and transcription factor networks. Int. J. Dev. Biol. 62, 195–205. 10.1387/ijdb.170298as, PMID: 29616729

[ref128] Sullivan C. H. Majumdar H. D. Neilson K. M. Moody S. A. (2019). Six1 and Irx1 have reciprocal interactions during cranial placode and otic vesicle formation. Dev. Biol. 446, 68–79. 10.1016/j.ydbio.2018.12.003, PMID: 30529252 PMC6349505

[ref129] Szabó A. Mayor R. (2018). Mechanisms of neural crest migration. Annu. Rev. Genet. 52, 43–63. 10.1146/annurev-genet-120417-031559, PMID: 30476447

[ref130] Szabó A. Theveneau E. Turan M. Mayor R. (2019). Neural crest streaming as an emergent property of tissue interactions during morphogenesis. PLoS Comput. Biol. 15:e1007002. 10.1371/journal.pcbi.1007002, PMID: 31009457 PMC6497294

[ref131] Theveneau E. Marchant L. Kuriyama S. Gull M. Moepps B. Parsons M.. (2010). Collective chemotaxis requires contact-dependent cell polarity. Dev. Cell 19, 39–53. 10.1016/j.devcel.2010.06.012, PMID: 20643349 PMC2913244

[ref132] Theveneau E. Steventon B. Scarpa E. Garcia S. Trepat X. Streit A.. (2013). Chase-and-run between adjacent cell populations promotes directional collective migration. Nat. Cell Biol. 15, 763–772. 10.1038/ncb2772, PMID: 23770678 PMC4910871

[ref133] Trainor P. A. (2013). Neural crest cells: Evolution, development and disease. Cambridge: Academic Press.

[ref134] Van Wijhe J. W. (1883). Ueber die Mesodermsegmente und die Entwickelung der Nerven des Selachierkopfes. Amsterdam: de Waal.

[ref135] Vandamme N. Berx G. (2019). From neural crest cells to melanocytes: cellular plasticity during development and beyond. Cell. Mol. Life Sci. 76, 1919–1934. 10.1007/s00018-019-03049-w, PMID: 30830237 PMC11105195

[ref136] Vega-Lopez G. A. Cerrizuela S. Aybar M. J. (2017). Trunk neural crest cells: formation, migration and beyond. Int. J. Dev. Biol. 61, 5–15. 10.1387/ijdb.160408gv, PMID: 28287247

[ref137] von Kupffer C. (1891). The development of the cranial nerves of vertebrates. J. Comp. Neurol. 1, 246–264. 10.1002/cne.910010306

[ref138] von Kupffer C. (1893). Studien zur vergleichenden Entwicklungsgeschichte des Kopfes der Kranioten. München: JF Lehmann.

[ref139] von Kupffer C. (1900). Zur Kopfentwicklung von Bdellostoma. Studien zur vergleichenden Entwicklungsgeschichte des Kopfes der Kranioten, Heft 4. München: Lehman.

[ref140] Washausen S. Knabe W. (2018). Lateral line placodes of aquatic vertebrates are evolutionarily conserved in mammals. Biol. Open 7:bio031815. 10.1242/bio.031815, PMID: 29848488 PMC6031350

[ref141] Wicht H. Northcutt R. G. (1995). Ontogeny of the head of the Pacific hagfish (*Eptatretus stouti*, Myxinoidea): development of the lateral line system. Philos. Trans. R. Soc. Lond. Ser. B Biol. Sci. 349, 119–134. 10.1098/rstb.1995.0098, PMID: 8668722

[ref142] Wicht H. Tusch U. (1998). “Ontogeny of the head and nervous system of myxinoids” in The biology of hagfishes. eds. Jørgensen J. M. Lomholt J. P. Weber R. E. Malte H. (Dordrecht, Netherlands: Springer), 431–451.

[ref143] Williams R. M. Candido-Ferreira I. Repapi E. Gavriouchkina D. Senanayake U. Ling I. T.. (2019). Reconstruction of the global neural crest gene regulatory network in vivo. Dev. Cell 51, 255.e257–276.e257. 10.1016/j.devcel.2019.10.003, PMID: 31639368 PMC6838682

[ref144] Wu C. -Y. Hooper R. M. Han K. Taneyhill L. A. (2014). Migratory neural crest cell αN-catenin impacts chick trigeminal ganglia formation. Dev. Biol. 392, 295–307. 10.1016/j.ydbio.2014.05.016, PMID: 24882712 PMC6933514

[ref145] Wu C. Y. Taneyhill L. A. (2019). Cadherin-7 mediates proper neural crest cell–placodal neuron interactions during trigeminal ganglion assembly. Genesis 57:e23264. 10.1002/dvg.23264, PMID: 30461190 PMC6932833

[ref146] Xu H. Dude C. M. Baker C. V. (2008). Fine-grained fate maps for the ophthalmic and maxillomandibular trigeminal placodes in the chick embryo. Dev. Biol. 317, 174–186. 10.1016/j.ydbio.2008.02.012, PMID: 18367162

[ref147] Yntema C. L. (1944). Experiments on the origin of the sensory ganglia of the facial nerve in the chick. J. Comp. Neurol. 81, 147–167. 10.1002/cne.900810204

[ref148] York J. R. Lee E. M. Mccauley D. W. (2019a). “The lamprey as a model vertebrate in evolutionary developmental biology” in Lampreys: Biology, conservation and control. ed. Docker M. F. (Dordecht, Netherlands: Springer), 481–526.

[ref149] York J. R. McCauley D. W. (2020a). Functional genetic analysis in a jawless vertebrate, the sea lamprey: insights into the developmental evolution of early vertebrates. J. Exp. Biol. 223:jeb206433. 10.1242/jeb.206433, PMID: 32034037

[ref150] York J. R. McCauley D. W. (2020b). The origin and evolution of neural crest cells. Open Biol. 10:190285. 10.1098/rsob.190285, PMID: 31992146 PMC7014683

[ref151] York J. R. Yuan T. Lakiza O. McCauley D. W. (2018). An ancestral role for semaphorin3F-neuropilin signaling in patterning neural crest within the new vertebrate head. Development 145:dev164780. 10.1242/dev.164780, PMID: 29980564

[ref152] York J. R. Yuan T. Zehnder K. McCauley D. W. (2017). Lamprey neural crest migration is snail-dependent and occurs without a differential shift in cadherin expression. Dev. Biol. 428, 176–187. 10.1016/j.ydbio.2017.06.002, PMID: 28624345

[ref153] York J. R. Zehnder K. Yuan T. Lakiza O. McCauley D. W. (2019b). Evolution of snail-mediated regulation of neural crest and placodes from an ancient role in bilaterian neurogenesis. Dev. Biol. 453, 180–190. 10.1016/j.ydbio.2019.06.010, PMID: 31211947

[ref154] Yuan T. York J. R. McCauley D. W. (2018). Gliogenesis in lampreys shares gene regulatory interactions with oligodendrocyte development in jawed vertebrates. Dev. Biol. 441, 176–190. 10.1016/j.ydbio.2018.07.002, PMID: 29981309

[ref155] Yuan T. York J. R. McCauley D. W. (2020). Neural crest and placode roles in formation and patterning of cranial sensory ganglia in lamprey. Genesis 58:e23356. 10.1002/dvg.23356, PMID: 32049434

[ref156] Zhang G. Cohn M. J. (2006). Hagfish and lancelet fibrillar collagens reveal that type II collagen-based cartilage evolved in stem vertebrates. Proc. Natl. Acad. Sci. U. S. A. 103:16829. 10.1073/pnas.0605630103, PMID: 17077149 PMC1636540

[ref157] Ziermann J. M. Diogo R. Noden D. M. (2018). Neural crest and the patterning of vertebrate craniofacial muscles. Genesis 56:e23097. 10.1002/dvg.23097, PMID: 29659153

[ref158] Zu Y. Zhang X. Ren J. Dong X. Zhu Z. Jia L.. (2016). Biallelic editing of a lamprey genome using the CRISPR/Cas9 system. Sci. Rep. 6:23496. 10.1038/srep23496, PMID: 27005311 PMC4804306

